# Anti-Angiogenic Activity of a Small Molecule STAT3 Inhibitor LLL12

**DOI:** 10.1371/journal.pone.0035513

**Published:** 2012-04-17

**Authors:** Hemant K. Bid, Duane Oswald, Chenglong Li, Cheryl A. London, Jiayuh Lin, Peter J. Houghton

**Affiliations:** 1 Center for Childhood Cancer, Nationwide Children's Hospital, Columbus, Ohio, United States of America; 2 College of Pharmacy, The Ohio State University, Columbus, Ohio, United States of America; 3 College of Veterinary Medicine, The Ohio State University, Columbus, Ohio, United States of America; The University of Kansas Medical Center, United States of America

## Abstract

**Background:**

Recent data indicate the Signal Transducer and Activator of Transcription 3 (STAT3) pathway is required for VEGF production and angiogenesis in various types of cancers. STAT3 inhibitors have been shown to reduce tumor microvessel density in tumors but a direct anti-angiogenic activity has not been described.

**Methodology/Principal Findings:**

We investigated the direct action of a small molecule inhibitor of STAT3 (LLL12) in human umbilical cord vascular endothelial cells (HUVECs) in vitro, in a Matrigel model for angiogenesis in vivo, and its antitumor activity in a xenograft model of osteosarcoma. LLL12 (100 nM) significantly inhibited VEGF-stimulated STAT3 phosphorylation in HUVECs, reduced their proliferation/migration and inhibited VEGF-induced tube formation. Morphologic analysis of LLL12 treated HUVECs demonstrated marked changes in actin/tubulin distribution and bundling. In *scid* mice, LLL12 reduced microvessel invasion into VEGF-infused Matrigel plugs by ∼90% at a dose of 5 mg/kg daily. Following a period of tumor progression (2 weeks), LLL12 completely suppressed further growth of established OS-1 osteosarcoma xenografts. Pharmacodynamic studies showed robust phosphorylated STAT3 in control tumors, whereas phospho-STAT3 was not detected in LLL12-treated OS-1 tumors. Treated tumors demonstrated decreased proliferation (Ki67 staining), and decreased microvessel density (CD34 staining), but no significant increase in apoptosis (TUNEL staining), relative to controls. Assay of angiogenic factors, using an antibody array, showed VEGF, MMP-9, Angiopoietin1/2, Tissue Factor and FGF-1 expression were dramatically reduced in LLL12-treated tumors compared to control tumors.

**Conclusions:**

These findings provide the first evidence that LLL12 effectively inhibits tumor angiogenesis both in vitro and in vivo.

## Introduction

Signal Transducer and Activator of Transcription 3 (STAT3) belong to the STAT family of transcription factors. Compelling evidence has now established that aberrant STAT3 is a molecular abnormality that has a critical role in the development and progression of not only adult but also some pediatric tumors [1]–[4]. In addition to its diverse biological functions including roles in cell proliferation, differentiation, apoptosis, inflammation, and oncogenesis, accumulating evidence suggests that STAT3 also plays an important role in cancer angiogenesis under both physiological and pathological situations [5]–[7]. There is accumulating evidence that STAT3 [8] is an important facilitator of tumor angiogenesis and its activation correlates with VEGF production in a variety of human cancers [9]. In addition to its effects on VEGF, STAT3 has been implicated as a facilitator of angiogenesis by other mechanisms. For example, it has recently been demonstrated that STAT3 regulates expression of both MMP-2 and MMP-9, important facilitators of both angiogenesis and metastasis [10]. It has been reported also that STAT3 is required for endothelial cell migration and microvascular tube formation [11]. These data implicate STAT3 as a key facilitator of angiogenesis beyond regulation of VEGF. Importantly, it has been demonstrated that STAT3 is critical for expression of HIF-1α, the best-documented transcriptional activator of VEGF and a wide variety of other angiogenic and invasive genes. STAT3 is thus an attractive molecular target for the development of novel anti-angiogenesis therapy. Several strategies have been already reported to block the action of STAT3 pathway, including antisense methods, inhibition of upstream kinases, phosphotyrosyl peptides or small molecule inhibitors [1], [12], [13]. In our study we used LLL12, a potent small molecule considered to block STAT3 dimerization and prevent STAT3 being recruited to the receptors and thus block JAK and possibly Src kinase-induced phosphorylation of STAT3. In the present study, we investigated the direct effect of LLL12 on angiogenesis in vitro and in vivo, and its antitumor activity against an established osteosarcoma xenograft model. Our findings clearly indicate that LLL12 directly inhibits tumor angiogenesis both in *in vitro* and *in vivo* models. *In vivo*, LLL12 significantly decreased growth of an osteosarcoma xenograft model. The antitumor activity of LLL12 was associated with decreased microvessel density, decreased tumor-associated angiogenic factors, and complete abrogation of phosphorylated STAT3 protein.

## Results

### LLL12 inhibits cellular viability/migration/invasion in human endothelial cells as well as viability of smooth muscle cells

**Figure 1:**
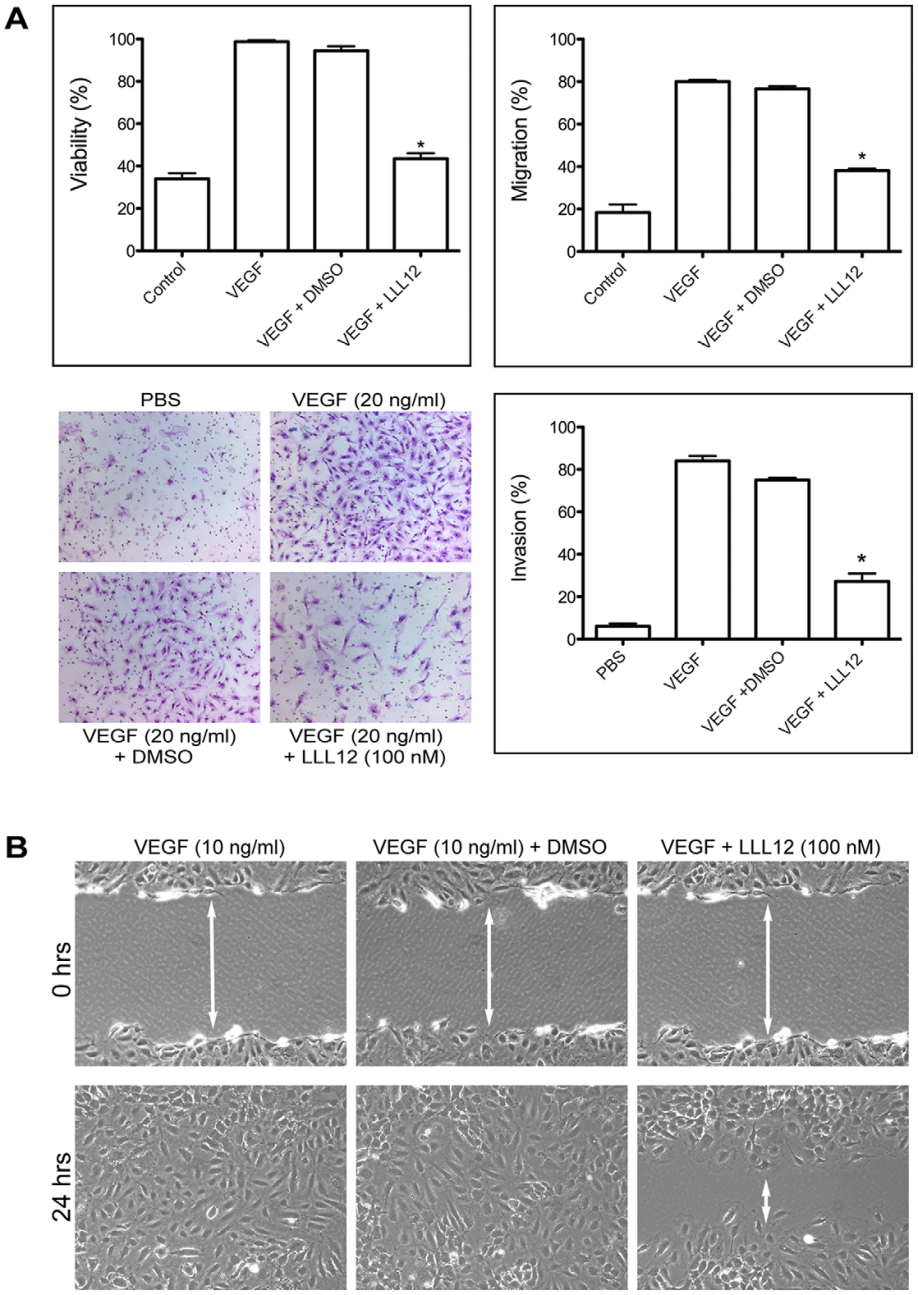
LLL12 inhibits angiogenesis in vitro. HUVECs were grown under serum-deficient conditions and stimulated with VEGF (10 ng/ml) in the absence or presence of LLL12 (100 nM). A. Proliferation/viability was determined after 2 days by Calcein AB staining. Migration was determined using the crystal violet assay as described in Materials and Methods as well as scratch assay/wound-healing. Invasion was determined using Matrigel coated membranes (photomicrographs show representative fields) and is quantified (bottom right panel). Each data set represents the mean ± SE for at least 3 independent experiments. B. To study the effect of LLL12 (100 nM) on the cell migration in HUVEC cells, wound-healing assays were carried out by allowing the cells to move to the scar region for 24 hours using VEGF (10 ng/ml) as a positive control in the presence and absence of LLL12 (original magnification ×40).

The small molecule inhibitor of STAT3, LLL12, has previously been shown to inhibit cellular proliferation and migration in several human malignant breast, pancreas and glioblastoma cells lines (IC_50_ concentrations ranging from 0.16–3.09 µM) [14], however inhibition of angiogenesis by this compound has not been investigated. To test in vitro anti-angiogenic activity of LLL12, we examined whether LLL12 inhibited proliferation of human umbilical vascular endothelial cells (HUVECs). Cells were stimulated with VEGF in the absence or presence of LLL12 and cell number determined after 2 days. As shown in **Figure 1A**, LLL12 inhibited proliferation in a concentration-dependent manner with >70% inhibition at 100 nM concentration. Two further assays demonstrated similar effects of LLL12 on invasion through Matrigel coated membranes, and in a wound-healing assay for migration (**Figure 1B**). Vascular smooth muscle cells (HASMCs), one of the major cell types of the vascular wall, play a critical role in the process of angiogenesis, under both physiological and pathophysiological conditions, including the cancer microenvironment. Therefore we performed a cell proliferation assay using HASMCs. LLL12 significantly inhibited proliferation of HASMCs at 100 nM concentration (data not shown).

### LLL12 inhibits VEGF-induced STAT3 phosphorylation and tube formation in HUVECs

**Figure 2:**
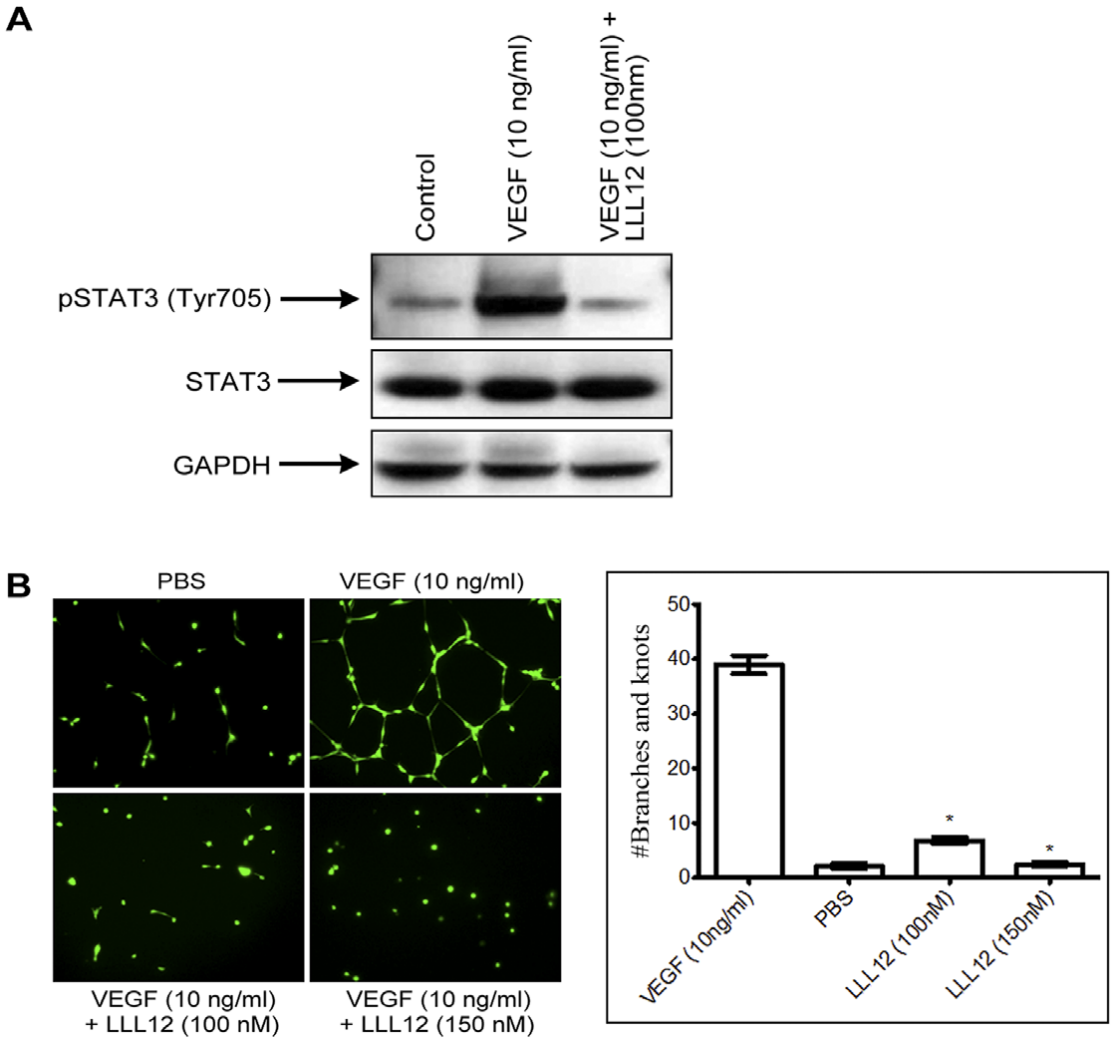
Inhibition of angiogenesis by LLL12 correlates with inhibition of STAT3 phosphorylation. A) Suppression of STAT3 phosphorylation by LLL12 in HUVEC cells after VEGF (10 ng/ml) stimulation. HUVEC cells were grown and stimulated with VEGF (10 ng/ml) for 10 mins and treated with LLL12 for 18–20 hrs. Total cellular protein was extracted and both total STAT3 and pSTAT3(Tyr705) expression was determined. GAPDH was used as a loading control. B) LLL12 inhibits HUVEC tube formation. HUVECs were grown in M200, and incubated with PBS or VEGF (10 ng/ml) for 18–20 hr in the absence or presence of LLL12 (100 nM). Tube formation was quantified as described in Materials and Methods.

The results above indicate that LLL12 inhibits HUVEC proliferation and migration at ∼100 nM. To determine whether this effect correlated with inhibition of STAT3 phosphorylation, HUVECs were grown under serum-deficient conditions and stimulated with VEGF (10 ng/ml) or PBS (control), and phosphorylated STAT3 determined after 18 hrs of LLL12 treatment. As shown in Figure 2A, VEGF-induced robust STAT3 phosphorylation in HUVEC cells, which supports the previous reports where in aortic macrovascular endothelial cells STAT3 is tyrosine phosphorylated in response to VEGF [15]. LLL12 treatment abolished VEGF-induced phosphorylation of STAT3 at drug concentrations that blocked VEGF-induced proliferation (**Figure 2A**). To examine whether LLL12 inhibited ‘capillary tube’ formation, HUVECs were grown under serum-deficient conditions and stimulated with VEGF or PBS (control). LLL12 at 100 nM concentration significantly inhibited formation of capillary-like structures (**Figure 2B**), indicating that signaling through STAT3 is necessary for VEGF-stimulated proliferation and tube formation of these endothelial cells.

### Inhibition of STAT3 disrupts the F-actin and microtubule cytoskeletal elements in HUVEC cells

**Figure 3:**
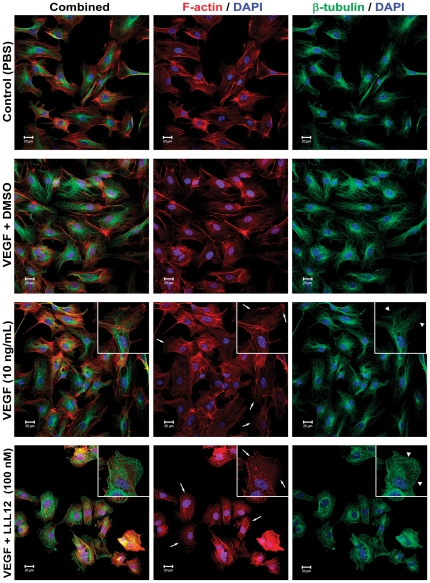
The STAT3 inhibitor, LLL12, induces cytoskeletal changes in cultured HUVEC cells. HUVEC cells cultured in 4-well chamber slides were treated with PBS, VEGF (10 ng/mL) alone, VEGF with DMSO or LLL12 (100 nM) for 18 hrs. The cultures were then probed using anti-β-tubulin primary antibodies (green), and F-actin was stained using phalloidin (red). White arrows highlight F-actin localization at the leading edge, while white arrowheads indicate the curling of microtubules at the cell periphery. 200× magnification. Slice depth = 1 µm. Scale bar = 20 µm. Inset 400× magnification.

Previous reports have shown that cytosolic STAT3 acts as a co-regulator of F-actin fiber [16] and microtubule [17] formation. Since LLL12 significantly reduced migration of HUVEC cells therefore, we hypothesized that disruption of lamellipodia formation at the leading edge, due to reduced Rac1 activity a downstream target in the STAT3 pathway, and microtubule breakdown at the trailing edge, may account for this phenomenon. To test this, HUVEC cultures were treated with VEGF alone, VEGF plus DMSO (drug vehicle) or VEGF with LLL12 (100 nM) for 18 hrs. After staining for F-actin and β-tubulin, HUVEC cultures treated with VEGF (10 ng/mL), or VEGF and the drug vehicle, DMSO, showed a greater number of cells than those treated with PBS (**Figure 3**). The F-actin in the control and VEGF-treated cultures produced thin, uniform fibers spanning the length of the cells, with a greater localization at the peripheral lamellipodia and intercellular junctions. The microtubules formed a dense lattice that emanated from the center of the cells, and extended to the periphery of the cells in a generally linear manner. However, in STAT3 inhibited cultures, the cells had a condensed, rounded morphology, compared to VEGF treated cultures. The F-actin had condensed into fewer fibers, and, most strikingly, was completely absent from the leading edges of the cells (white arrows, Figure 3). The microtubule structures were additionally affected by the LLL12 treatment. As highlighted by the arrowheads in Figure 3, β-tubulin staining still showed that the microtubules emanated from the nuclear region of the HUVEC cells, but at the periphery, they curled over, unable to extend to the leading edge.

### LLL12 is a potent Inhibitor of Angiogenesis in Vivo

**Figure 4:**
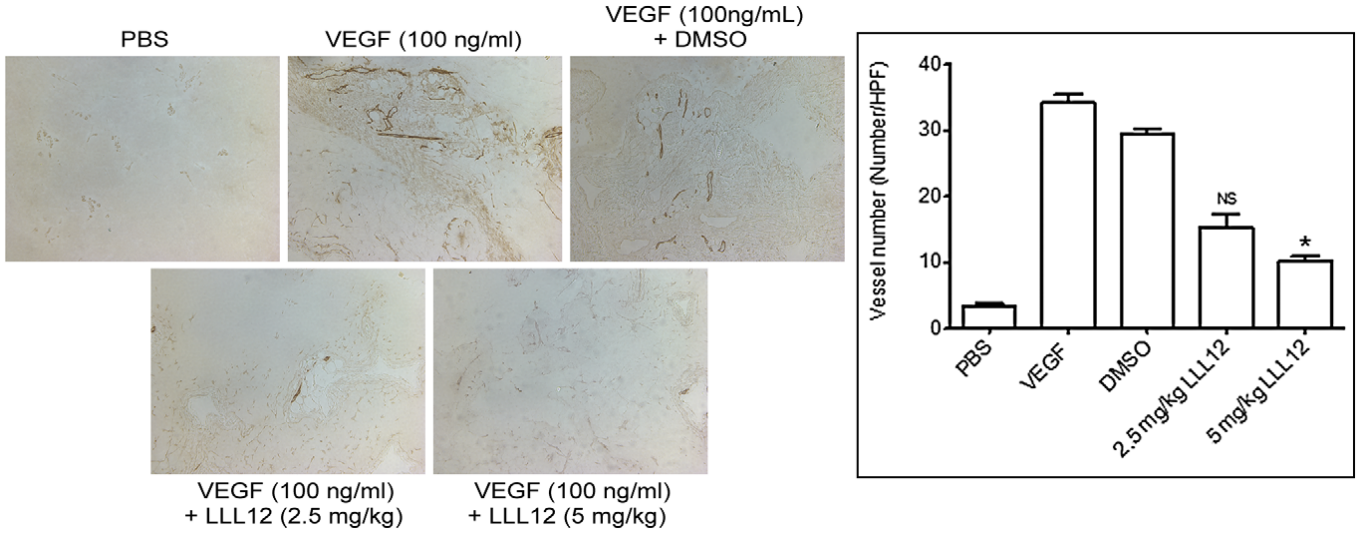
LLL12 inhibits angiogenesis in mice. Matrigel plugs containing PBS (Control), or VEGF (100 ng/ml) were implanted subcutaneously in mice that were or were not treated with LLL12 (5 mg/kg daily). The Matrigel plugs were excised on day 7, fixed with formalin and 5-µm sections were stained for CD34 staining. The numbers of CD34 positive vessels per high power field (HPF, magnification, 200×) were counted for each experimental condition. Results are mean (n = 4) ± SE. *P<0.05; versus VEGF alone.

Because in HUVECs LLL12 was observed to be both anti-migratory and proliferative *in vitro* (Figures. 1, and 2), its effect on angiogenesis *in vivo* was investigated using a Matrigel plug assay. To directly test the anti-angiogenic activity of LLL12 *in vivo*, mice were implanted subcutaneously with Matrigel plugs infused with PBS or VEGF. Mice were treated with LLL12 (2.5 and 5 mg/kg by I.P administration) immediately after implantation of the plug and once daily for 7 days. Plugs were excised at day 7 and angiogenesis quantified as described in Materials and Methods. VEGF increased the number of vessels detected in Matrigel plugs by >10-fold over that in PBS infused (control) plugs. LLL12 reduced vessel formation at 2.5 mg/kg and significantly (by ∼75%) at 5 mg/kg dose level compared to controls (**Figure 4**).

### LLLL12 inhibits tumor angiogenesis and tumor growth in Osteosarcoma Xenografts

**Figure 5:**
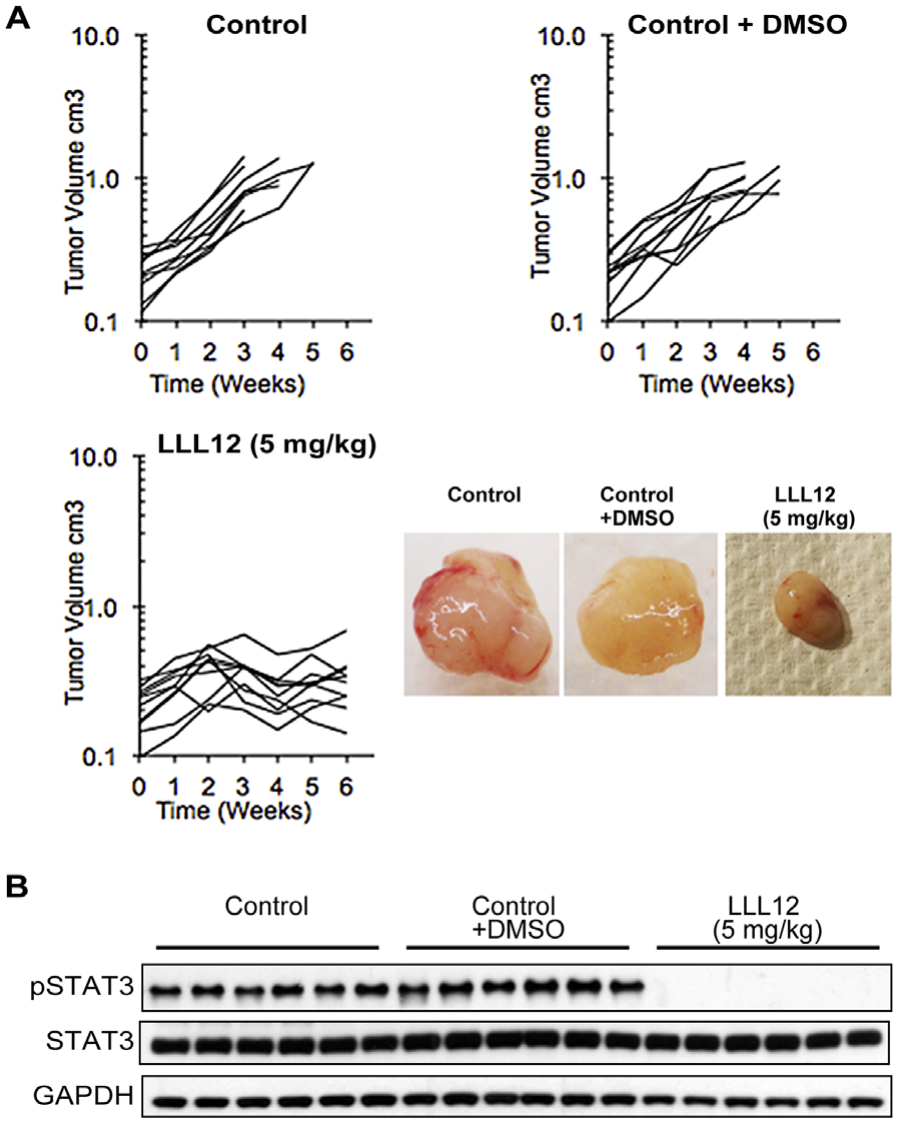
LLL12 inhibits tumor growth *in vivo* by inhibition of STAT3. A, LLL12 inhibits tumor growth in osteosarcoma xenograft mice. OS-1 tumors were transplanted into 6-week-old CB17SC *scid−/−* female mice. After tumors grew to ∼130 mm^3^, mice were randomized to receive no treatment (control), DMSO or LLL12 (5 mg/kg/d) for a planned six weeks. LLL12 inhibited tumor growth as measured by tumor volume. Representative tumors at the termination of each group are shown. B. Western blot showing STAT3, and p-STAT3 level in six independent tumors from each treatment group. LLL12 completely blocks pSTAT3 levels with compassion to control and DMSO control group.

We examined the inhibitory function on tumor growth by LLL12 using an osteosarcoma xenograft model. Growth of control (no treatment) or vehicle treated OS-1 xenografts was highly reproducible (**Figure 5A**). Mice were terminated when tumors grew to a volume four-fold greater than the volume at the start of treatment, usually after 3 to 4 weeks, and tumors were snap-frozen for biochemical determinations. LLL12 was administered at 5 mg/kg (based on the dose having the greatest anti-angiogenic effects) was well tolerated with no mortality. In LLL12 treated mice there was a period of continued growth (2 weeks) followed by complete tumor stasis for the remaining 4 weeks of treatment. As shown in Figure 5A, after a 6 weeks the mean tumor volume of LLL12-treated group were significantly less than that of control or DMSO group at time of termination (P<0.01).

**Figure 6:**
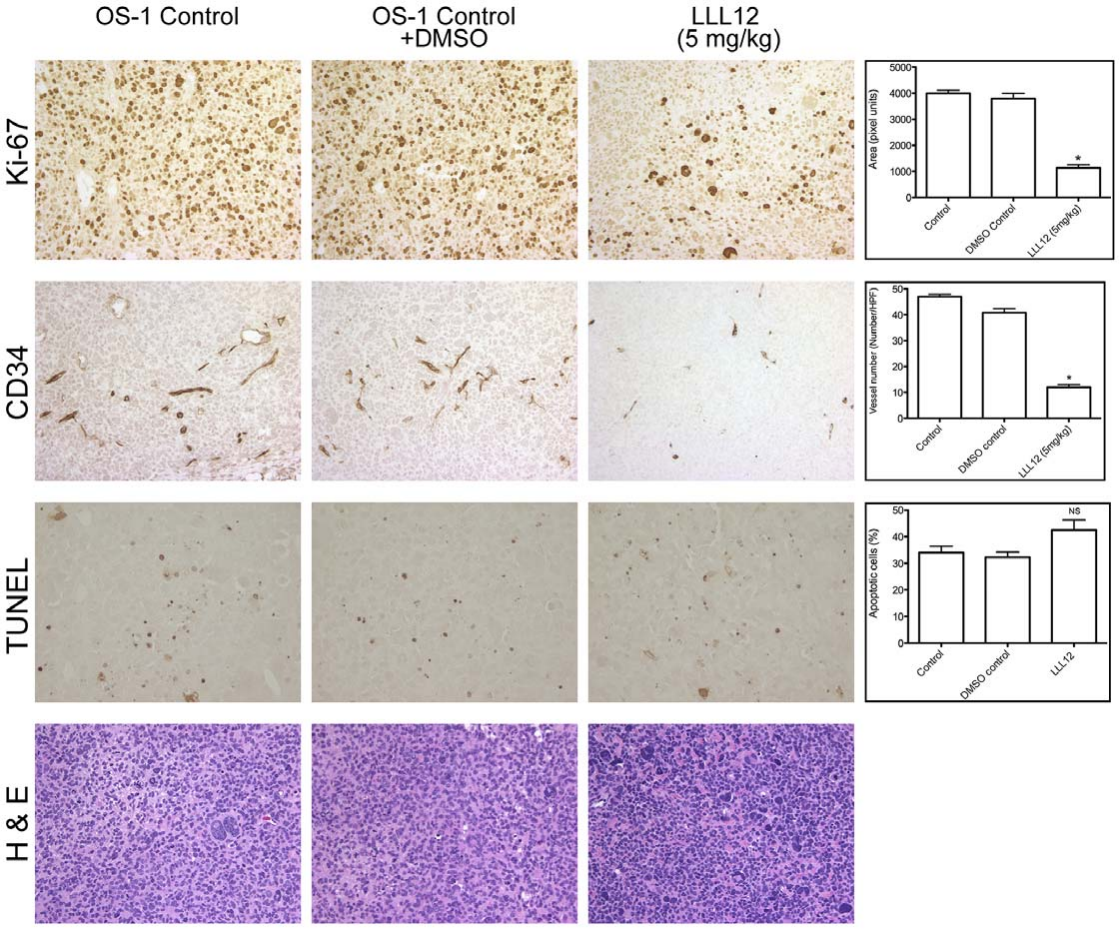
LLL12 inhibits angiogenesis in OS-1 xenografts. Analysis of vascularity and tumor cell proliferation: Tumors were harvested at completion of the study and examined by hematoxylin and eosin staining. Vascularity of osteosarcoma tumor xenografts in mice was evaluated by CD34 related antigen staining (brown) for endothelial cells, and Ki 67 for proliferation. Apoptotic cells were identified by TUNEL staining. There is a significant effect of LLL12 as compared to vehicle control on tumor vasculature as well as cell proliferation (quantified by staining with anti–Ki-67) in osteosarcoma xenografts, but no significant change in apoptosis in drug treated tumors. All values are expressed as mean plus or minus SEM. *P<.01 by Student *t* test.

To examine the pharmacodynamic effects of LLL12, total and phospho-STAT3, Ki67 and CD34 staining as well as apoptosis (TUNEL) were determined in control, vehicle alone (DMSO) and LLL12 treated tumors at the end of treatment or when tumors reached 4-times the initial volume (controls). As shown in **Figure 5B**, robust phospho-STAT3 was detected in all control or DMSO treated tumors, in contrast after 6 weeks of treatment with LLL12 no phospho-STAT3 could be detected, although total STAT3 was unchanged compared to controls. To evaluate the effect of LLL12 on tumor angiogenesis, 5-µm tumor sections were stained with anti-CD34 antibody. The average vessel number in LLL12–treated group was dramatically decreased compared to control or DMSO treated groups (**Figure 6A**), indicating that LLL12 significantly inhibits tumor angiogenesis. Also there was la lower frequency of proliferating cells (Ki67-positive) in LLL12 treated tumors compared to control and DMSO treated groups (**Figure 6B**). However, LLL12 treatment did not increase the incidence of TUNEL-positive cells, suggesting the action of this drug against OS-1 xenografts is largely cytostatic (**Figure 6C**).

### LLL12 inhibits not only VEGF but also other important factors for new vessel formation in OS-1 xenografts

**Figure 7:**
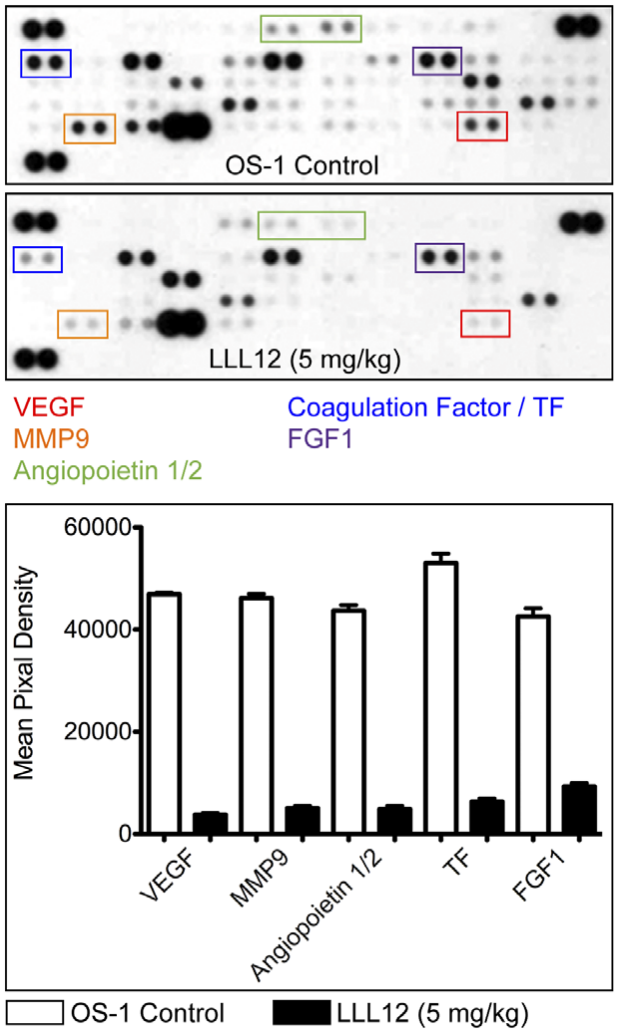
LLL12 downregulates several angiogenic factors. LLL12 induced changes in angiogenic factors in OS-1 xenografts were determined using a Proteome profiler antibody array as described in Materials and Methods. The effect of LLL12 treatment is quantified in the histogram, untreated tumors (−), tumors from mice receiving LLL12 (+), 5 mg/kg).

Previous reports indicate that in addition to its effects on VEGF, STAT3 facilitates angiogenesis by other mechanisms. To examine whether targeting STAT3 by LLL12 inhibits not only VEGF but also other critical angiogenic factors in osteosarcoma tumors, we examined the levels of 55 angiogenesis-associate proteins using a human angiogenesis array. We analyzed the array data in osteosarcoma tumors. Antibody array studies of the osteosarcoma tumor lysates were derived from control and treated groups discussed above. Relative to control OS-1 xenografts, LLL12 treated tumors showed a dramatic decrease of VEGF, MMP-9, Angiopoietin, tissue factor (TF) and FGF-1 (**Figure 7**), critical regulators of angiogenesis (Michael et al 1991). We used the Pediatric Preclinical Testing Program (PPTP) expression data set [18] for pediatric tumor xenografts (http://pptp.nchresearch.org/data.html) to examine the expression of human angiogenic genes in osteosarcomas relative to other pediatric solid tumor and leukemia models. Osteosarcoma xenografts express high levels of VEGF-A, angiopoetin 1, Tissue Factor and MMP9, relative to leukemia xenografts. Expression of angiopoeitin 1 was generally higher in osteosarcoma xenografts than in most other pediatric solid tumors, whereas among the osteosarcoma xenografts FGF1 was expressed most highly in the OS-1 model.

### LLL12 directly inhibits growth of sarcoma cell lines

We examined direct effects of LLL12 on sarcoma cell proliferation. Tumor cells were exposed to LLL12 (1–10^4^ nM) for approximately four cell divisions and viability was determined by Alamar Blue staining. Of interest the human osteosarcoma line, OS-17 and the canine osteosarcoma cell line, Abrams) were more sensitive than either Rh30 (rhadomyosarcoma) or EW8 (Ewing sarcoma) human cell lines, **Table 1**.

**Table 1:** Sensitivity of sarcoma cell lines to LLL12.

Cell Line	IC50 (µM) ± St. Dev
EW8	2.3±0.31
Rh30	1.4±0.25
OS17	0.23±0.03
Abrams	0.044±0.002

## Discussion

LLL12 is a novel small molecule allosteric inhibitor of STAT3, thought to bind STAT3 monomers at the tyrosine 705-phosphorylation site and to prevent dimerization and activation. Previous work has established that LLL12 inhibits proliferation of various cancer cells in vitro, and tumor growth of both breast and glioblastoma xenograft models [14]. Moreover, LLL12 induces apoptosis in medulloblastoma and glioblastoma cells and was also able to inhibit colony formation, wound healing and decreased IL-6 and LIF secretion [19]. Antisense STAT3 oligonucleotide or STAT3 inhibitors, other than LLL12, have been shown to reduce microvessel density in tumor models [20], [21]. However, the mechanism for these anti-angiogenic effects has not been investigated. Our current work shows that at concentrations of drug that abrogate STAT3 phosphorylation, LLL12 blocks angiogenesis, and suppresses tumor vasculature in osteosarcoma tumors.

The direct effect of LLL12 suppressing proliferation of HIVEC and HASMCs was shown at low concentrations of drug that completely suppressed VEGF-stimulation of STAT3 phosphorylation. LLL12 also potently inhibited HUVEC migration and invasion at this concentration, suggesting that STAT3 signaling is intimately involved in these processes. LLL12 exerted marked effects on both F-actin fibers and microtubules in HUVECs. In treated cells, F-actin had condensed into fewer fibers, and was completely absent from the leading edges of the cells. Similarly, microtubule structures emanated from the nuclear region, but at the periphery, they curled over, unable to extend to the leading edge. These observations substantiate that STAT3 is a necessary modulator of Rac1 activity at the leading edge of cells, and that RhoA stabilization of already formed actin fibers was largely unaffected. They further show that without F-actin at the periphery, the cells are unable to grow and/or migrate, and that the structural microtubules cannot extend to the leading edges, further compounding the effects of STAT3 inhibition. Together, these effects account for the reduction of HUVEC cell migration shown previously.

In vivo, VEGF stimulated vascular cell invasion ∼10-fold over that of PBS-infused Matrigel. Daily treatment with LLL12, starting immediately after Matrigel plug implantation, showed a significant, dose-dependent, inhibition of CD34-positive cells into the VEGF-infused Matrigel plugs, confirming that the effects seen in vitro could be recapitulated at tolerable dose levels of drug in vivo.

We subsequently investigated the activity of LLL12 against a human osteosarcoma xenograft model, OS-1. Treatment with LLL12 was started against established xenografts (median volume 200 mm^3^). Interestingly, tumor growth was maintained at rates similar to control tumors (no treatment or drug vehicle only) for two weeks. Subsequently, further treatment resulted in complete tumor growth inhibition. The results for LLL12 differ from previous results with angiogenesis inhibitors, cedirinib and sunitinib [5], [22], or sorafenib (unpublished data). Cedirinib and sorafenib induced complete growth stasis from initiation of treatment, whereas sunitinib significantly retarded the rate of OS-1 growth from start of treatment. The reason behind this relatively slow onset of tumor growth retardation is not known, but may relate to rapid clearance of LLL12 from plasma (unpublished data), and slow accumulation of drug into tumor tissue. However, analysis of phospho-STAT3 in tumors at the end of 6 weeks treatment showed complete abrogation of signal compared to robust phosphor-STAT3 detected in control tumors at the time the mice were euthanized. The rate of proliferation (Ki67 positive cells) of OS-1 tumors was significantly reduced, as was microvessel density, consistent with an angiogenic effect of LLL12. In contrast, there was no significant change in the frequency of apoptotic cells as judged by TUNEL staining, suggesting the effect of LL12 is largely cytostatic in this tumor model.

Our data indicate that STAT3 inhibition effectively suppresses growth of OS-1 osteosarcoma xenografts. LLL12 appears to have both direct and indirect effects on angiogenesis. Firstly, LLL12 inhibits proliferation of vascular elements by blocking the response to VEGF in vitro and in vivo. LLL12 inhibited VEGF-stimulated phosphorylation of STAT3 at a concentration similar to that blocking proliferation, migration and capillary tube formation in HUVECs, suggesting that STAT3 signaling is important in these processes. Secondly, LLL12 reduced tumor-associated angiogenic factors (VEGF, MMP-9, angiopoetin, TF and FGF1), probably as a direct consequence of STAT3 inhibition in tumor cells. Whether inhibition of STAT3 in OS-1 tumor cells directly inhibits proliferation is not known. OS-1 grows only as a xenograft, and there is no isogenic cell line model in vitro. However, LLL12 does directly inhibit growth of human carcinoma cell lines with IC_50_ concentrations in the 1–5 µM range [14]. LLL12 potently inhibited proliferation of OS17 and also the canine osteosarcoma model. In contrast, the other sarcoma cell lines (Rh30 and Ew8) were 6–10-fold less sensitive. It is thus likely that inhibition of STAT3 signaling by LLL12 inhibits tumor growth through a combination of its direct and indirect effects on angiogenesis and direct inhibitory effect on tumor cell proliferation.

## Materials and Methods

### Reagents

M200, M231, media, fetal bovine serum (FBS) and Calcein Alamar Blue (Calcein AB) were purchased from Invitrogen (Carlsbad, CA). Low serum growth supplement (LSGS) and Smooth Muscle Growth Supplement (SMGS) was obtained from Cascade Biologics Inc (Portland Oregon). Endothelial Tube formation assay kits were from Cell Biolabs, Inc. (San Diego, CA). Growth factor reduced Matrigel for *in vivo* experiments and precoated Matrigel inserts for invasion assays were purchased from BD Biosciences (Palo Alto, CA). Anti-CD34 antibody (ab27448) was from Abcam (Cambridge, MA, USA). LLL12 (5-Hydroxy-9,10-dioxo-9,10-dihydroanthracene-1-sulfonamide) was synthesized in the Laboratory of Dr Pui-Kai Li (College of Pharmacy, The Ohio State University). The powder was dissolved in sterile dimethylsulfoxide (DMSO) to make a 5 mg/ml stock solution. Aliquots of the stock solution were stored at −20°C.

### Cell Culture

Human umbilical vein endothelial cells (HUVEC) were obtained from the American Type Culture Collection (ATCC). All experiments were done using endothelial cells between passages 3 and 8. HUVECs were maintained in endothelial cell growth medium M200 (Invitrogen) in high glucose supplemented medium with 15% FBS, endothelial cell growth supplements (LSGS Medium, Cascade Biologics), and 2 mM glutamine at 37°C with 5% CO_2_. All cells were maintained as sub confluent cultures and split 1∶3, 24 hours before use. Primary human aortic smooth muscle cells (HASMC) were obtained from Cascade biologicals and all experiments were done using HASMC between passages 3 and 8. HASMC were maintained in M231 (Invitrogen). To induce differentiation of normal human vascular smooth muscle cells, Medium 231 was supplemented with Smooth Muscle Differentiation Supplement (Invitrogen) and cells were incubated at 37°C with 5% CO_2_.

### Western blotting

Cell lysis, protein extraction and immunoblotting were as described previously (Kurmasheva et al 2009,). We used primary antibodies to glyceraldehyde-3-phosphate dehydrogenase (GAPDH), pSTAT3 (Tyr 705) and STAT3 (Cell Signaling). Immunoreactive bands were visualized by using Super Signal Chemiluminiscence substrate (Pierce) and Biomax MR and XAR film (Eastman Kodak Co.). Fonly ifteen µL of total volume and 20 µg protein sample was resolved on a 4–12% SDS-polyacrylamide gel. Proteins were transferred to a PVDF membrane and immune-detection was performed with specific primary antibodies.

### Endothelial Cell Tube Formation Assay

The Endothelial Tube Formation Assay (CBA200, Cell Biolabs Inc., San Diego, CA, USA) was used in addition to the HUVEC proliferation assay. The ECM gel was thawed at 4°C and mixed to homogeneity using cooled pipette tips. Cell culture plates (96-well) were bottom-coated with a thin layer of ECM gel (50 µl/well), which was left to polymerize at 37°C for 60 min. HUVEC (2–3×10^4^ cells) were stimulated with VEGF in 150 µl medium and added to each well on the solidified ECM gel. Culture medium was added to each well in the presence or absence of LLL12. The plates were incubated at 37°C for 12–18 hr and the endothelial tubes were quantified using a fluorescent microscope after staining with Calcein AM. Three microscope fields were selected at random and photographed. Tube forming ability was quantified by counting the total number of cell clusters (knots) and branches under a 4× objective and four different fields per well. The results are expressed as mean fold change of branching compared with the control groups. Each experiment was performed at least three times.

### Cell viability/proliferation assay

HUVECs and HASMCs were seeded on 6-well plates at a density of approximately 1×10^5^ cells/well in their respective media. Cells were treated with 100 nM of LLL12 one day after seeding. After two days, Calcein AB was added directly into culture media at a final concentration of 10% and the plates were incubated at 37°C. Optical density (OD) was measured spectrophotometrically at 540 and 630 nm with at 3–4 h after adding Calcein AB. As a negative control, Calcein AB was added to medium without cells. To determine sensitivity to LLL12 of sarcoma cell lines, cells were exposed to increasing concentrations of LLL12 for approximately four cell divisions, depending on the rate of proliferation of each cell line.

### Quantitative migration assay

HUVECs were grown in M200 containing LSGS until 40–50% confluent. Cells were washed with PBS, trypsinized for 5–10 min, collected with 0.2% FBS and centrifuged at 300 g for 5 min. Cells were then resuspended with 0.2% FBS and counted using a Beckman Coulter Z2. A volume of 400 µl of this mix containing 5×10^5^ cells was placed on to Boyden Chambers (8 µm pore) inserts with and without LLL12 (100 nM) in 24 well plates with 500 µl of M200. VEGF (R and D Systems) in 1% BSA was added to a final concentration of 10 ng/ml in the lower chambers as a chemo-attractant. Cells were pretreated with antibody for 30 min in suspension, then placed in the chambers and incubated at 37°C 5% CO_2_ for 18–24 hrs. The Boyden chamber porous membranes were then blotted and fixed with 3.7% formaldehyde containing 0.05% crystal violet for 30 min. After repeated washes with distilled water the membranes were air-dried. The migrated cells on the bottom side of the membranes were collected by scraping the bottom of the chamber with a Q-tip, which was subsequently placed into a 1.5 ml eppendorf tube and incubated in 80% methanol to extract the dye. The remainder of the cells on top of the membrane and within the Boyden chamber, were separately incubated in 80% methanol, shaken at 500 rpm for 30 min, and the extracted dye measured at 570 nm. Migration was quantified using the ratio of the migrated cells over the total cells (migrated plus remaining cells) to determine the fraction of migrating cells in each individual experiment. Experiments were performed in duplicate on multiple occasions as described in the figure legends.

### Cell migration assay (in vitro wound-healing assay)

HUVEC migration was monitored using the wound-healing assay described by Thaloor et al. [23]. Briefly, 3×10^4^ cells/well/ml were seeded in 24-well plates using M200 medium with LSGS. After cells had attached and form a monolayer on plates completely, we scraped the cells with same width using yellow tip. After scraped, the cells were washed with PBS and were incubated with the medium containing VEGF (10 ng/ml)) with or without LLL12 (100 nM). Width was photographed at different time intervals (0 and 24 hours) with a magnification of 40× with a microscopic camera system (Leitz Diavert microscope, Leica, Bensheim; AxioCam, Carl Zeiss, Gottingen, Germany).

### Invasion Assay

The Matrigel invasion assays were carried out using Matrigel precoated inserts (BD Bioscience) following the manufacturer's instructions. Six hundred µl of M200 medium with or without VEGF (20 ng/ml, R&D Systems) was placed in the lower wells. Proliferating HUVECs (4×10^5^ cells/ml) were pretreated with LLL12 (100 nM) and 100 µl of cell suspension was loaded into each of the upper wells. The chambers were incubated for 18–20 hr at 37°C. After incubation, the inserts were removed, and the non-invading cells on the upper surface were removed with a cotton swab. The cells on the lower surface of the membrane were fixed in 100% methanol for 15 minutes, air-dried, and stained with Diff-Quik stain for 2 min. The cells were counted in six individual high-power fields for each membrane condition under a light microscope. Assays were performed in triplicate for each treatment group and the results were expressed as migrated cells per field for each condition [24].

### Vascularization of Matrigel™ Plugs *in vivo*

To further characterize anti-angiogenetic properties of LLL12 *in vivo*, we performed murine Matrigel plug experiments. PBS was used as a negative control, and VEGF (100 ng/mL) as a positive control. Matrigel was injected subcutaneously into CB17SC *scid* ^−/−^ female mice, forming semi-solid plugs. Animals received treatment of LLL12 (5 mg/kg) I.P. intraperitoneal (i.p.) injection immediately after the Matrigel injection and daily for 7 days. On day 7, plugs were excised under anesthesia, fixed in PBS-buffered 10% formalin containing 0.25% glutaraldehyde, and were processed for H & E and Masson's Trichrome staining. Vascular identity of the infiltrating cells was established with CD34 immunostaining. The regions containing the most intense area of neovascularization (“hotspots”) were chosen for analysis. Eight hotspots were identified for each Matrigel or tumor section. The ImagePro Plus analysis system (Media Cybernetics Inc, Bethesda, MD) was used to quantify the percentage of area occupied by the vessel-like structures in each field. The mean ± SE from each group were compared. The negative control was obtained by tissue staining with secondary antibody only.

### In Vivo Tumor Growth Inhibition Studies

CB17SC-F *scid−/−* female mice (Taconic Farms, Germantown, NY) were used to propagate subcutaneously implanted OS-1 osteosarcoma tumors [25], [26]. All mice were maintained under barrier conditions and experiments were conducted using protocols and conditions approved by the institutional animal care and use committee of the appropriate consortium member. Mice were randomized into groups of 10 when tumors were 100 to 200 mm^3^. LLL12 was administered by intraperitoneal (i.p.) injection at indicated dose levels every day, for 6 weeks. Tumor volumes were determined as previously described [26].

### Ethics Statement

All animal experiments were conducted in accordance with institutional animal care and use committee of the The Research Institute at Nationwide Children's Hospital approved protocols, designed to minimize the numbers of mice used and to minimize any pain or distress. The named institutional review board or ethics committee specifically approved this study.

### Immunohistochemistry (IHC)

For IHC, fixed tumors were sectioned at 5-µm then dewaxed and soaked in alcohol. After microwave treatment in antigen unmasking solution (Vector Laboratory, Burlingame, CA, USA) for 10 min, endogenous peroxidase activity was inactivated by incubating in 3% hydrogen peroxide (H_2_O_2_) for 15 min and sections were incubated with primary antibody in phosphate-buffered saline at 4°C overnight. After washing with phosphate-buffered saline (PBS), immuno-staining was performed using the Vectastain Universal Quick Kit and DAB Peroxidase Substrate Kit (Vector Laboratories, Burlingame, CA, USA) according to the manufacturer's instructions. Antiserum was omitted in the negative control. The number of cells staining positive was counted by a blinded observer in 5 random 40× fields and treated *versus* controls compared (Student t test). Images were obtained with an Olympus AX70 fluorescence microscope and Spot v2.2.2 (Diagnostic Instruments, Sterling Heights, MI) digital imaging system. Apoptosis was detected in deparaffinized tumor sections by TdT-mediated dUTP nick-end labeling (TUNEL) assay using the in situ cell death detection kit (Cat no. Cat. No. L00297, GenScript USA Inc) according to manufactures instructions.

### Confocal Microscopy

HUVEC cells were grown in Lab-Tek 4-well chamber slides (Nunc/Thermo Scientific) to approximately 60% confluence, and treated with PBS, VEGF (10 ng/mL) DMSO with VEGF, or 100 nM LLL12 for 18 hrs with VEGF. The cultures were fixed in cold 4% paraformaldehyde for 20 mins at 4°C, and then cells were permeabilized in 0.1% Triton X-100 (Sigma-Aldrich). The cells were probed using 10 µg/mL mouse anti-human β-Tubulin I primary antibody (Sigma-Aldrich, clone TUB 2.1) overnight at 4°C, and then stained using 10 µg/mL goat anti-mouse Alexa Fluor® 488 conjugated F(ab')_2_ (Invitrogen) for 1 hr. at RT. F-actin in the cells was stained at RT for 1 hr. using 4 U/mL Alexa Fluor® 633 conjugated phalloidin (Invitrogen). The cells nuclei were then stained by incubating with 300 nM 4′,6-diamidino-2-phenylindole (DAPI) dilactate (Invitrogen) for 5 mins at RT. Samples were washed 4 times in PBS after each step during processing. After mounting a coverslip, 1024×1024 pixel images were taken using a Zeiss Axiovert 710 confocal microscope with a slice depth of 1 µm. Image processing was performed using the Zeiss Image Browser software package.

### Human Angiogenesis Array

Proteome profiler antibody array (R & D systems; Cat no ARY007, Minneapolis, MN) was used according to manufacturer's instructions to detect the relative levels of expression of 55 angiogenesis related proteins in control and treated tumors. After blocking the membranes 300 µg of protein from the tumor tissue lysate from control and LLL12 treated groups were added and incubated for overnight at 4°C. Next day the membranes were washed and streptavidin-HRP was added or 30 minutes. Immunoreactive signals were visualized by using Super Signal Chemiluminiscence substrate (Pierce) and Biomax MR and XAR film (Eastman Kodak Co.). Array data on developed X-ray film was quantified by scanning the film using Biorad Molecular Image Gel Doc™ XR+ and analyze the data using Image Lab™ software.

### Statistical analysis

Significance of correlations was done using GraphPad Prism software. Unpaired *t* tests were used for all analyses assuming Gaussian populations with a 95% confidence interval. Data are presented as mean ± SE. Differences were analyzed with the Student *t* test, and significance was set at *P* less than <0.05.
